# Supplementary material for: Research on the concentration, potential and mission of science and technology innovation in China
Source: PLoS One. 2021 Oct 11;16(10):e0257636. doi: 10.1371/journal.pone.0257636 (PMC8504756; doi:10.1371/journal.pone.0257636)
Supplement: Attachment — Submitted filename: Response to Reviewer 3.docx [file pone.0257636.s002.docx]

Response to Reviewer 3 Comments

Dear Reviewer:

First of all, thank you very much for your feedback! These are excellent suggestions, and we wholeheartedly agree with your points. Here are our responses in terms of your feedback:

**Point 1:**

I think the justification for using TOPSIS and Entropy should be explained more in the introduction section.

**Response 1:**

Thank you very much for your suggestions. We have supplemented the related theories and analysis of Topsis and entropy, thank you for your contribution.

**Point 2:**

I think the paper should be improved by adding a new section or subsection to present other MCDM and weighting methods which can be used for the assessment process; like COPRAS (COmplex PRoportional Assessment), WASPAS (Weighted Aggregates Sum Product Assessment), SECA (Simultaneous Evaluation of Criteria and Alternatives), MEREC (MEthod based on the Removal Effects of Criteria) and EDAS (Evaluation based on Distance from Average Solution).

**Response 2:**

Thank you very much for your help with our article. We have re-added the source of weights and analysis methods, combined with your comments to strengthen the interpretation of the evaluation process. Finally, we emphasize the suitability and uniqueness of this research method. thanks for your help

**Point 3:**

Acronyms and abbreviations should be defined at their first mention. Please check them in the manuscript. For example: Technique for Order of Preference by Similarity to Ideal Solution (TOPSIS). The equations should be numbered and organized. I think a sensitivity analysis should be made based on changing criteria weights to show the stability of results.

**Response 3:**

Thanks for your guidance. We adjusted the corresponding content one by one to make the article structure more appropriate.

**Point 4:**

I think a sensitivity analysis should be made based on changing criteria weights to show the stability of results. A conclusion section should be added to present final discussion, concluding remarks and suggestions for future research.

**Response 4:**

We re-use the analytic hierarchy process to get new weights. Combining the comparison of the two weights and conducting sensitivity analysis, it is found that the location changes of the provinces have not changed, which shows the stability and validity of the results. At the same time, we have added the conclusions and prospects to make research more valuable.

* Again, we thank you for your helpful inputs! We believe the new considerations and major revisions should better show the paper's rationale.

Take care, and Thank you so much.
